# The impact of the Boko Haram insurgency in Northeast Nigeria on childhood wasting: a double-difference study

**DOI:** 10.1186/s13031-018-0136-2

**Published:** 2018-01-24

**Authors:** Gillian Dunn

**Affiliations:** 1 City University of New York (CUNY) School of Public Health, 55 West 125th Street, New York, NY 10027 USA; 2 Present Address: Hawaii Pacific University, 45-045 Kamehameha Hwy, Kaneohe, HI 96744 USA

**Keywords:** Malnutrition, Wasting, Weight-for-height, Conflict, Boko Haram, Nigeria, Double-difference, Difference-in-difference, Demographic and Health Surveys

## Abstract

**Background:**

This research examines the relationship between violent conflict and childhood wasting in Northeast Nigeria, where residents have been subjected to fighting between the Nigerian government and Boko Haram - an extremist Islamist movement - since 2009.

**Methods:**

Using two Demographic and Health Surveys from before and after the Boko Haram insurgency started, a double-difference (difference-in-difference) approach is used to assess the impact of the conflict on mean weight-for-height z-scores and the likelihood of wasting.

**Results:**

Results suggest that if children exposed to the conflict had not been exposed, their mean weight-for-height z-score would be 0.49 standard deviations higher (*p* < 0.001) than it is, increasing from − 0.74 to − 0.25. Additionally, the likelihood of wasting would be 13 percentage points lower (mean z-statistic − 4.2), bringing the proportion down from 23% to 10%.

**Conclusion:**

Descriptive evidence suggests that poor child health outcomes in the conflict areas of Northeast Nigeria may be due to disruptions to social services and increased food insecurity in an already resource poor area. Although other unidentified factors may contribute to both conflict and wasting, the findings underscore the importance of appropriate programs and policies to support children in conflict zones.

**Electronic supplementary material:**

The online version of this article (10.1186/s13031-018-0136-2) contains supplementary material, which is available to authorized users.

## Background

Conflict impacts child health through multiple pathways. Community and household resources may be diminished as funds are diverted away from social services, prices for food and other commodities rise, and fear or physical obstacles prevent caregivers from pursuing livelihood activities [1, 2]. Infrastructure such as health facilities, markets, water supply and sewage systems, and roads may be damaged or otherwise inaccessible [1, 2]. Supply chains for food imports and essential medicines are often disrupted [1, 2]. Populations may be forced to leave a conflict zone, which could expose them to inadequate shelter, water, sanitation, and food and deprive them of livelihoods [1, 2]. Health care personnel may leave the area while the most vulnerable households may be unable to do so [2].

Most deaths due to conflict - particularly for children - are not from direct causes such as war-related trauma, but are attributable to the conditions that were already the main causes of death before the conflict (severe malnutrition, diarrheal disease, acute respiratory infections, etc.) [3]. Malnutrition is of particular interest in the study of child health and conflict because a) it is a contributing condition in about 45% of child deaths worldwide and b) it is sensitive to disruptions commonly found in war zones such as increased food insecurity [3, 4]. In the Democratic Republic of Congo, malnutrition was cited by respondents as an underlying or primary cause of death in 8.1% of deaths in non-conflict areas and 10.9% of deaths in conflict areas [5]. In Angola, higher rates of malnutrition were found in conflict-affected areas and among assumed supporters of the opposition [6].

The objective of this study is to explore whether conflict has had a deleterious effect on the nutritional status of children in Northeast Nigeria by examining the counterfactual – what the status of children would have been had they not been exposed to conflict. Here, nutritional status is confined to wasting (low weight-for-height), as it is the preferred measure in acute emergencies because weight is sensitive to sudden changes in food availability and infections [1, 6, 7]. It is hypothesized that that children not exposed to conflict will display better nutritional status than those exposed to conflict, both in terms of mean weight-for-height and the likelihood of being wasted.

### Nigeria and Boko Haram

**Fig. 1:**
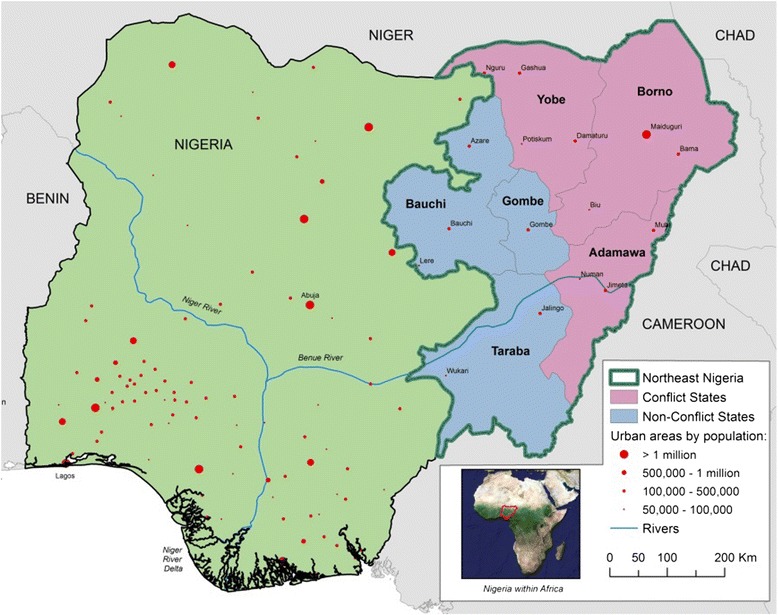
Nigeria with the conflict and non-conflict states of the Northeast

Nigeria is Africa’s most populous country and among its most diverse with over 400 ethnolinguistic groups [8]. The country is affected by several conflicts based on overlapping ethnic, religious, political and regional divisions including over resources in the Niger Delta, Christian-Muslim divides in the middle of the country, and most recently, the rise of Islamist groups in the north, most importantly, Boko Haram [8]. Boko Haram (‘Western education is a sin’) was founded around 2002 in Maiduguri, the capital of Borno state and largest city in Northeast Nigeria (Fig. 1) [9]. At least at its inception, the main tenet among its followers was regime change in Nigeria as they believe democratic and secular rule is in contradiction to *Shariah* [9].

In July 2009, the Boko Haram uprising began in Bauchi and spread to other northern states, leaving hundreds of followers, Nigerian law enforcement officers, and civilians dead [10]. The following year, attacks in the Northeast and other parts of the country including bombings, mass shootings, and executions began to rise [10].

**Fig. 2:**
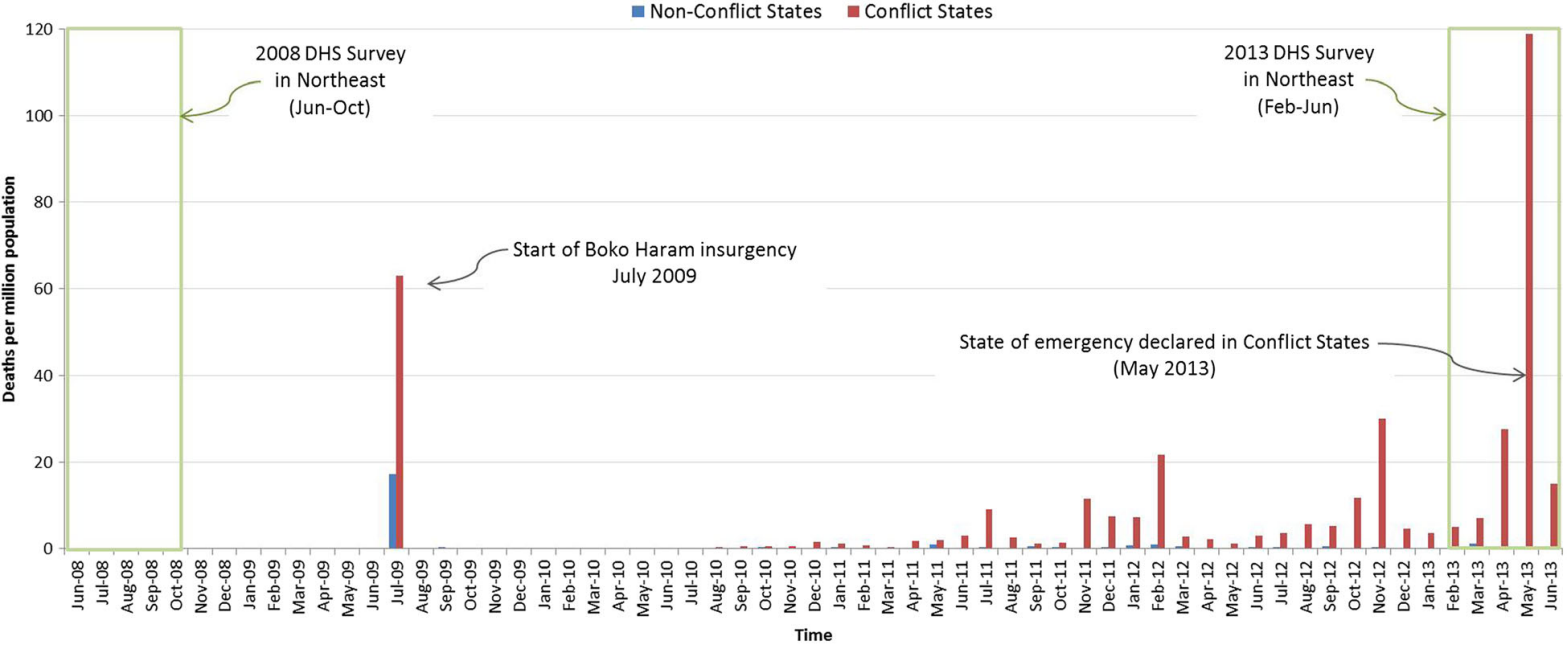
Timeline of violent deaths attributed to events involving Boko Haram in Northeast Nigeria. Data from Nigeria Watch [40]

In May 2013, the president declared a state of emergency in the states of Borno, Yobe, and Adamawa [10]. For this study, these states are defined as “conflict affected” and are compared to Bauchi, Gombe, and Taraba (the “non-conflict states”). People in the non-conflict states have certainly been affected by the crisis, but the entire population of Borno, Yobe, and Adamawa states are considered directly impacted by the Boko Haram insurgency [11]. Fig. 2 shows a timeline of violent deaths attributed to events involving Boko Haram, which serve as a proxy for the intensity of the conflict. The timeline also shows July 2009 as a hard date for the start of the crisis in its current violent form [12]. This serves as the demarcation between pre-intervention and post-intervention for this study.

## Methods

### Data

This study uses data from two Demographic and Health Surveys (DHS), one from 2008 and the other from 2013 (Fig. 2) [13]. The DHS are large, nationally-representative household surveys which are comparable across countries and time periods [13]. DHS uses a two-stage sampling methodology. The country is first stratified by geographic (usually administrative) regions crossed with urban or rural designation. In each stratum, enumeration areas determined by the most recent census are used. In the first stage, a number of primary sampling units are selected from the enumeration areas in each stratum. The household lists in these areas are updated, and a fixed number of households are selected. All household members within a specific group (relevant here is all women age 15–49) in the selected households are chosen for the survey [14]. Those women with children age 0–59 months are asked about the health and care of their children and the children are measured and weighed [13, 14]. Sampling weights are provided which are used in combination with the strata and primary sampling units to obtain weighted observations.

The outcome of interest is wasting, which here is measured both as a continuous variable using z-scores for weight-for-height (WHZ) and as a binary outcome (moderately/severely wasted or not). Moderate wasting is defined as between − 3 and − 2 z-scores below the median of the WHO child growth standards and severe wasting is defined as below − 3 z scores [15].

In addition to exposure to conflict (living in areas under a government-declared state of emergency), explanatory covariables include:

Environmental factors:

- The month of the interview to control for seasonal effects of the surveys.
- Altitude, which may determine livelihoods and remoteness of populations.
- Urban/rural designation as per Esri’s base map layer “populated places”, which is derived from national censuses [16].
- Urban population rank as per Esri’s base map layer “populated places”, which includes detail on population size [16].

Household characteristics:

- Wealth as determined by an index which accounts for the assets and services available to a household [17]. For this analysis, a binary variable was created for whether the household was in the bottom two wealth quintiles (poorest households) or not.
- Water source and toilet type, classified as either “improved” or “unimproved” as per the WHO/UNICEF classification scheme [18]. Water and sanitation are part of the wealth index, but showed little collinearity for this dataset (VIF = 1.15–1.35).
- Number of people in household, which can have a positive effect on child health (many caregivers) or negative (stretched resources). For this reason, this term was also squared.
- Number of children under five and this term squared. Similar to the variable above, a household with many children may show pooled resources, but can also mean at least some of the children do not get the attention or resources they need [19].

Caregiver (child’s mother and mother’s partner) characteristics:

- Occupation, which is classified here as either in subsistence/own activities or wage employment.
- Educational attainment, here as a binary variable of no formal education or some education (primary, secondary, or higher).
- Mother’s religion, classified as Muslim or other (Christian or traditionalist).

Child characteristics:

- Age in months. The term is also squared to account for a possibly non-linear relationship.
- Sex, as several studies show slight differences in malnutrition outcomes between boys and girls [20, 21].
- Birth order and the term squared as higher birth orders may be protective up to a certain limit due to the experience of the mother and the help older siblings may provide in basic childcare [19].

### Double-difference (DD) analysis

Double-difference (also known as difference-in-difference) analysis is a methodology often used to estimate the causal effects of policies or programs [22]. It is a quasi-experimental design which makes use of before and after groups, but without random assignment [23]. In DD analysis, first a time- and population-specific intervention is identified. Then the difference in outcomes after and before the intervention for those affected by the intervention are compared to the difference in outcomes after and before the intervention among those not affected by the intervention.

The DD approach is most frequently used within a linear regression model with a continuous outcome variable to which covariates may be added. In this study, the intervention is conflict and the model may be written as:

$$ y={\beta}_0+{\beta_1}^{\ast } Conflict+{\beta_2}^{\ast } Period+{\beta_3}^{\ast}\left({Conflict}^{\ast } Period\right)+{\beta_k}^{\ast}\left( environmental, household, caregiver, and child covariates\right)+\varepsilon $$

where the outcome variable *y* is the mean z-score, *Conflict* is a dummy variable coded 0 for conflict states and 1 for non-conflict states (since we are interested in what the outcome would have been for the exposed group had they *not* been exposed), and *Period* is a dummy variable coded 0 for 2008 and 1 for 2013. The main coefficient of interest is *β*_*3*_ - the interaction term of *Conflict* and *Period* - which is the estimate of the effect of the double difference. *Β*_*k*_ represents the coefficients for covariates included in the model and *ε* is the error term.

In addition to any differences in mean z-scores, it is important to know if there are any changes in the likelihood that a child will be in the most vulnerable group – moderately or severely malnourished. For this, logistic regression is preferred; however, calculating the marginal effects of an interacted term such as the DD coefficient is not straightforward in non-linear models [24]. Therefore, the user-written Stata command *inteff*, which computes the correct marginal effect for the interaction term as well as the standard errors and z-statistic, is used [24].

The fundamental assumption of DD analysis is that of common trends [25]. Here, common trends means that if the Boko Haram insurgency had not occurred, the difference in malnutrition prevalence in all the Northeastern states would be constant over time. If this assumption is not fulfilled, any estimation of the causal effect of the conflict will be biased. Common trends is tested by examining earlier data together with the time period of interest. Here, DHS surveys from 1990 and 2003 were examined with the 2008 and 2013 surveys. The proportions of wasting adjusted for month of interview to account for seasonal differences in the surveys were calculated [26]. This was conducted in Stata with the user-written command *svypxcat* [27].

The 1990 and 2003 DHS surveys were at the zone (e.g., Northeast) level, so the GPS coordinates of the sampling points were used to determine states. These earlier surveys also used a different nutrition reference standard; therefore the raw height and weight data were used to calculate the z-scores with the WHO standards using the Stata user-written command *zscore06* [28].

## Results

### Population description

Descriptive statistics for the 2008 and 2013 populations are shown in Tables 1 and 2. Mean WHZ increased in the non-conflict states by 0.14 between 2008 and 2013, while decreasing in the conflict states by 0.40. While wasting declined in the non-conflict states by 10%, the percentage increased in the conflict states from 18 to 23%. The conflict states are more urbanized, mostly due to the presence of Maiduguri. The percentage of households in the wealthiest three quintiles grew, but unevenly; 10% in conflict states, but with no proportional change in the non-conflict states. Similarly, the average increase in access to improved water sources and toilet types from 2008 to 2013 was from 34 to 45%, but with a larger increase in the conflict states (water 18%, toilet 12%) vs. non-conflict states (water 10%, toilet 3%). Education rates are low in the Northeast; 72% of mothers and 60% of their partners have no formal education. About 85% of the mothers are Muslim.

**Table 1:** Mean weight-for-height z-scores in conflict and non-conflict states of Northeast Nigeria, 2008 and 2013

Variable	2008		2013	
	Non-Conflict	Conflict	Non-Conflict	Conflict
	Mean (95% CI)	Mean (95% CI)	Mean (95% CI)	Mean (95% CI)
WHZ	−0.74 (−0.75 - -0.73)	−0.35 (−0.36 - -0.34)	− 0.60 (− 0.62 - -0.58)	−0.75 (− 0.77 - -0.73)

**Table 2:** Percentage of wasting, environmental, household, caregiver, and child characteristics in conflict and non-conflict states of Northeast Nigeria, 2008 and 2013

Variable	2008		2013	
	Non-Conflict	Conflict	Non-Conflict	Conflict
	Percent (95% CI)	Percent (95% CI)	Percent (95% CI)	Percent (95% CI)
Wasting				
No	73 (72–74)	82 (81–83)	83 (81–84)	77 (75–79)
Yes	27 (26–28)	18 (17–19)	17 (16–19)	23 (21–25)
Month of interview				
Feb – Apr	–	–	61 (59–63)	65 (63–67)
May – Jul	39 (37–41)	42 (41–44)	39 (37–41)	35 (33–37)
Aug – Oct	61(59–63)	58 (56–59)	–	–
Altitude (meters)				
92–250	17 (16–18)	12 (11–13)	13 (12–14)	11 (10–12)
251–499	59 (57–61)	77 (76–78)	66 (64–68)	77 (75–79)
501–999	23 (22–24)	9 (8–10)	15 (14–16)	8 (7–9)
1000–1562	2 (1.6–2.4)	2 (1.6–2.4)	6 (5–7)	4 (3–5)
Urban/Rural				
Rural	91 (90–92)	82 (81–83)	91 (90–92)	77 (75–79)
Urban	9 (8–10)	18 (17–19)	9 (8–10)	23 (21–25)
Urban population				
50–100,000	1 (0.7–1.3)	3 (2–4)	–	–
100–500,000	8 (7–9)	8 (7–9)	9 (8–10)	11 (10–12)
> 1 million	–	7 (6–8)	–	13 (12–14)
Not urban	91 (90–92)	82 (81–83)	91 (90–92)	77 (75–79)
Wealth				
Wealthiest 3 quintiles	28 (27–29)	31 (30–32)	28 (26–30)	38 (36–40)
Poorest 2 quintiles	72 (71–73)	69 (68–70)	72 (70–74)	62 (60–64)
Water source				
Unimproved	71 (70–72)	64 (63–65)	61 (59–63)	46 (44–48)
Improved	29 (28–30)	35 (34–36)	39 (37–41)	53 (51–55)
missing	1 (−0.03–0.08)	–	1 (0.6–1.4)	1 (0.6–1.4)
Toilet type				
Unimproved	67 (66–69)	61 (60–62)	64 (62–66)	48 (46–50)
Improved	32 (31–33)	39 (38–40)	35 (33–37)	51 (49–53)
missing	1 (−0.03–0.08)	1 (0.7–1.3)	1 (0.06–1.4)	1 (0.6–1.4)
No. people in household				
2–5	31 (28–31)	29 (28–30)	28 (26–30)	38 (36–40)
6–8	32 (31–33)	35 (34–36)	31 (29–33)	32 (30–34)
9–43	37 (35–39)	36 (35–37)	41 (39–43)	30 (28–32)
No. under fives in household				
1	18 (17–19)	18 (17–19)	17 (16–19)	23 (21–25)
2	38 (36–40)	37 (36–38)	35 (33–37)	40 (38–42)
3–9	43 (41–45)	45 (43–47)	47 (45–49)	36 (34–38)
missing	1 (0.7–1.3)	1 (0.7–1.3)	1 (0.6–1.4)	1 (0.6–1.4)
Mother’s occupation				
Wage employment	47 (45–49)	35 (34–36)	50 (48–52)	32 (30–34)
Subsistence/own activities	53 (51–55)	64 (63–65)	48 (46–50)	67 (65–69)
missing	1 (0.7–1.3)	1 (0.7–1.3)	1 (0.6–1.4)	–
Partner’s occupation				
Wage employmnet	44 (42–46)	47 (45–49)	47 (45–49)	49 (47–51)
Subsistence/own activities	54 (52–56)	51 (49–53)	52 (50–54)	50 (47–51)
missing	3 (2–4)	2 (1.6–2.4)	1 (0.6–1.4)	1 (0.6–1.4)
Mother’s education				
Some education	29 (28–30)	25 (24–26)	31 (29–33)	28 (26–30)
No formal education	71 (70–72)	75 (74–76)	69 (67–71)	72 (70–74)
Partner’s education				
Some education	39 (37–41)	33 (32–34)	45 (43–47)	35 (33–37)
No formal education	57 (55–59)	65 (64–66)	53 (51–55)	64 (62–66)
missing	4 (3–5)	1 (0.7–1.3)	2 (1.4–2.5)	2 (1.4–2.5)
Mother’s religion				
Other	20 (19–21)	13 (12–14)	17 (16–19)	9 (8–10)
Muslim	79 (78–80)	87 (86–88)	83 (81–84)	91 (90–92)
Child’s age (months)				
0–5	14 (13–15)	12 (11–13)	12 (11–13)	10 (9–11)
6–11	11 (10–12)	10 (9–11)	11 (10–12)	11 (10–12)
12–23	20 (19–21)	19 (18–20)	21 (19–23)	2 (1–3)
24–59	56 (54–58)	59 (58–60)	57 (55–59)	59 (57–61)
Child’s sex				
Female	49 (47–51)	51 (49–53)	49 (47–51)	49 (47–51)
Male	51 (49–53)	49 (47–51)	51 (49–53)	51 (49–53)
Birth order				
1st - 2nd	32 (31–33)	28 (27–29)	30 (28–32)	37 (35–39)
3rd - 4th	27 (26–28)	29 (27–29)	26 (24–28)	28 (26–30)
5th - 17th	41 (39–43)	44 (42–46)	44 (42–46)	36 (34–38)
No. of obs. (weighted)	3810	4168	2462	2572

### Common trends

**Fig. 3:**
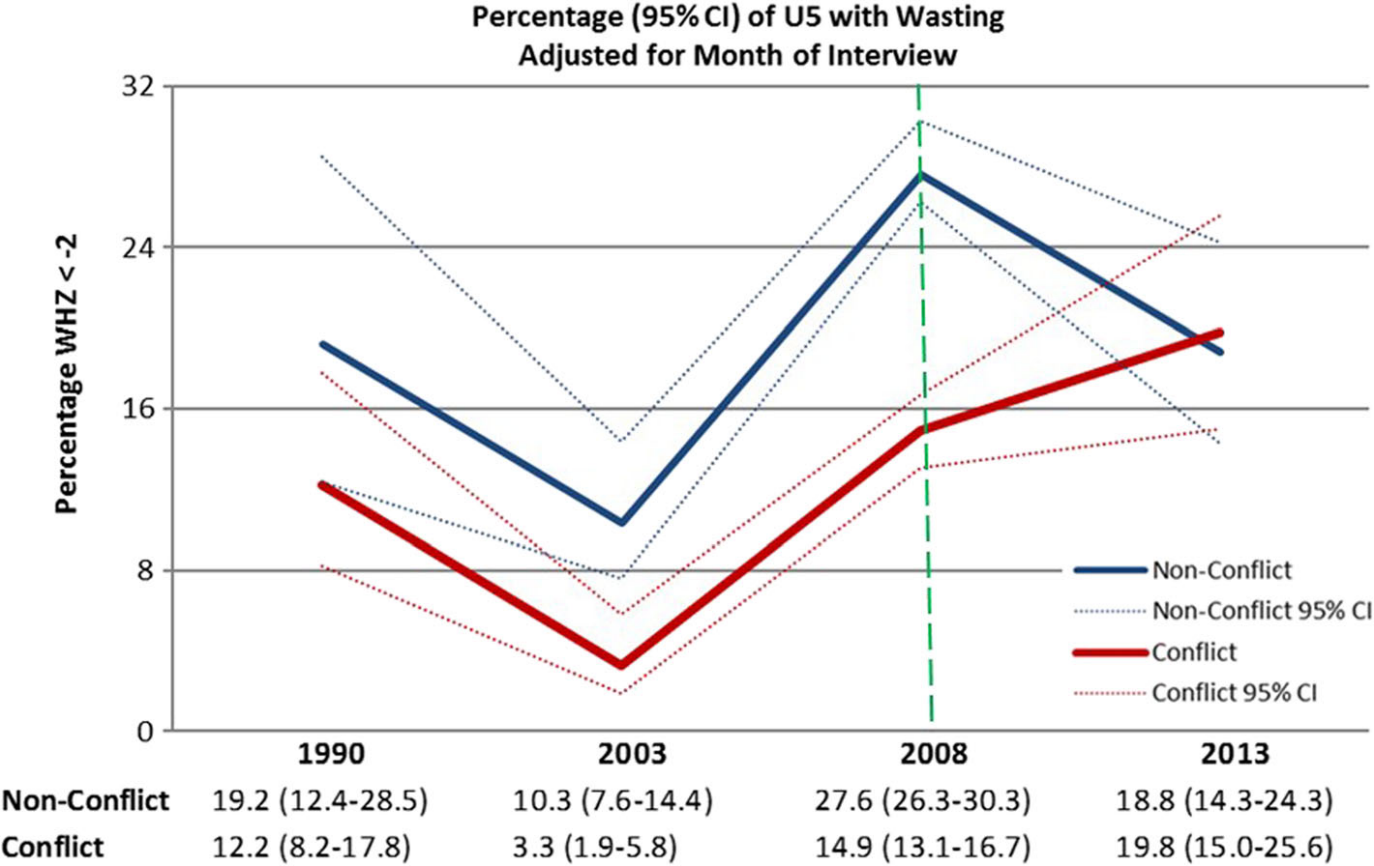
Trends in wasting in Northeast Nigeria’s conflict and non-conflict states. Percentages are adjusted for month of household interview. Dashed green line is the time point between the 2008 and 2013 surveys where the trends diverge

There is no statistical test for common trends analysis, but visualization such as in Fig. 3 is helpful for discerning patterns. Here, wasting trends are similar before the start of the conflict and then diverge after the start of the conflict, thus fulfilling the common trends requirement.

### Weight-for-height z-scores

The results of the linear regression for weight-for-height z-scores are shown in Table 3. Model 1 is without covariates and Model 2 has all environmental, household, and child covariates. Model 3 has select covariates based on their significance in Model 2 (*p* < 0.05). The main coefficient of interest is “double-difference”, which is highly significant in all the models. The results suggest that, *ceteris paribus*, if children who were exposed to the Boko Haram insurgency had not been exposed, their mean WHZ would be 0.49 standard deviations higher than it is (*p* < 0.001). More variance can be explained with the addition of covariates to Model 1. On average, z-scores increase if the interview was conducted in the rainy season compared to the dry season. Altitude has a small, but significant effect with z-scores decreasing slightly for every meter gain in elevation. Urban/rural classifications were not statistically significant, although the size of urban center was. Wealth was marginally significant, but the other household variables were not. Children whose mothers had no formal education had a mean WHZ 0.13 SD lower than those whose mothers had some formal education. Children of Muslim mothers had a mean WHZ 0.29 SD lower than children of other religions. Z-scores decreased slightly for every additional month of age and on average, boys had slightly lower z-scores than girls.

**Table 3:** Results of linear regression for weight-for-height z-scores. Model 1 has no covariates, Model 2 includes all environmental, household, caregiver, and child covariates, Model 3 has select covariates based on significance in Model 2 (*p* < 0.05)

	WHZ Model 1 (no covar.)			WHZ Model 2 (all covar.)			WHZ Model 3 (select covar.)		
	Coeff.	*p*-value	Std. Err.	Coeff.	p-value	Std. Err.	Coeff.	p-value	Std. Err.
States									
Conflict states	ref			ref			ref		
Non-conflict states	−0.370	0.001	0.110	−0.481	0.000	0.107	−0.473	0.000	0.105
Period									
2008	ref			ref			ref		
2013	−0.371	0.000	0.091	−0.273	0.049	0.138	−0.265	0.056	0.138
Double-Difference									
States * Period = 0	ref			ref			ref		
States * Period = 1	0.463	0.001	0.141	0.486	0.000	0.119	0.486	0.000	0.120
Month									
February				0.294	0.008	0.110	0.278	0.013	0.110
March				0.252	0.000	0.067	0.246	0.000	0.065
April				0.240	0.001	0.069	0.234	0.001	0.067
May				0.223	0.001	0.066	0.205	0.002	0.064
June				0.323	0.000	0.077	0.319	0.000	0.076
July				0.387	0.000	0.087	0.394	0.000	0.084
August				0.338	0.001	0.097	0.327	0.001	0.095
September				0.125	0.220	0.101	0.114	0.257	0.101
October				0.412	0.000	0.114	0.380	0.001	0.111
Altitude (m)									
Altitude				−0.001	0.036	0.001	−0.001	0.027	0.000
Altitude squared				0.000	0.010	0.000	0.000	0.007	0.000
Urban/Rural									
Rural				ref					
Urban				−0.094	0.745	0.288			
Urban population									
50–100,000				ref			ref		
100–500,000				0.293	0.330	0.301	0.259	0.401	0.308
> 1 million				0.805	0.009	0.304	0.721	0.020	0.309
Not urban				–	–	–	0.103	0.724	0.290
Wealth									
Wealthiest 3 quintiles				ref					
Poorest 2 quintiles				0.140	0.067	0.076			
Water source									
Unimproved				ref					
Improved				0.020	0.754	0.064			
Toilet type									
Unimproved				ref					
Improved				−0.010	0.882	0.068			
No. people in household									
No. people				0.017	0.351	0.018			
No. people squared				−0.001	0.208	0.000			
No. under fives in household									
No. under fives				0.002	0.981	0.079			
No. under fives squared				−0.003	0.770	0.010			
Mother’s occupation									
Wage employment				ref					
Subsistence/own activites				0.017	0.767	0.058			
Partner’s occupation									
Wage employment				ref					
Subsistence/own activities				−0.042	0.501	0.062			
Mother’s education									
Some education				ref			ref		
No formal education				−0.132	0.019	0.056	−0.133	0.011	0.052
Partner’s education									
Some education				ref					
No formal education				−0.077	0.180	0.057			
Mother’s religion									
Other				ref			ref		
Muslim				−0.252	0.010	0.097	−0.292	0.001	0.089
Child’s age (months)									
Child’s age				−0.018	0.003	0.006	−0.019	0.001	0.005
Child’s age squared				0.000	0.000	0.000	0.000	0.000	0.000
Child’s sex									
Female				ref			ref		
Male				−0.107	0.007	0.039	−0.090	0.017	0.038
Birth order									
Birth order				0.030	0.349	0.031			
Birth order squared				−0.002	0.372	0.003			
Intercept Constant	−0.366	0.000	0.059	−0.404	0.160	0.287	−0.271	0.460	0.366
R-squared	0.008			0.056			0.054		
F-test	0.0001			0.0000			0.0000		

The weight-for-height models have several significant coefficients, but low r-squared terms, indicating that the data have high variability, but that the explanatory covariables still provide information about the outcome. Thus, while these models have little predictive precision, the objective of the analysis – to determine whether there are before-after effects due to conflict – is accomplished.

### Wasting

**Fig. 4:**
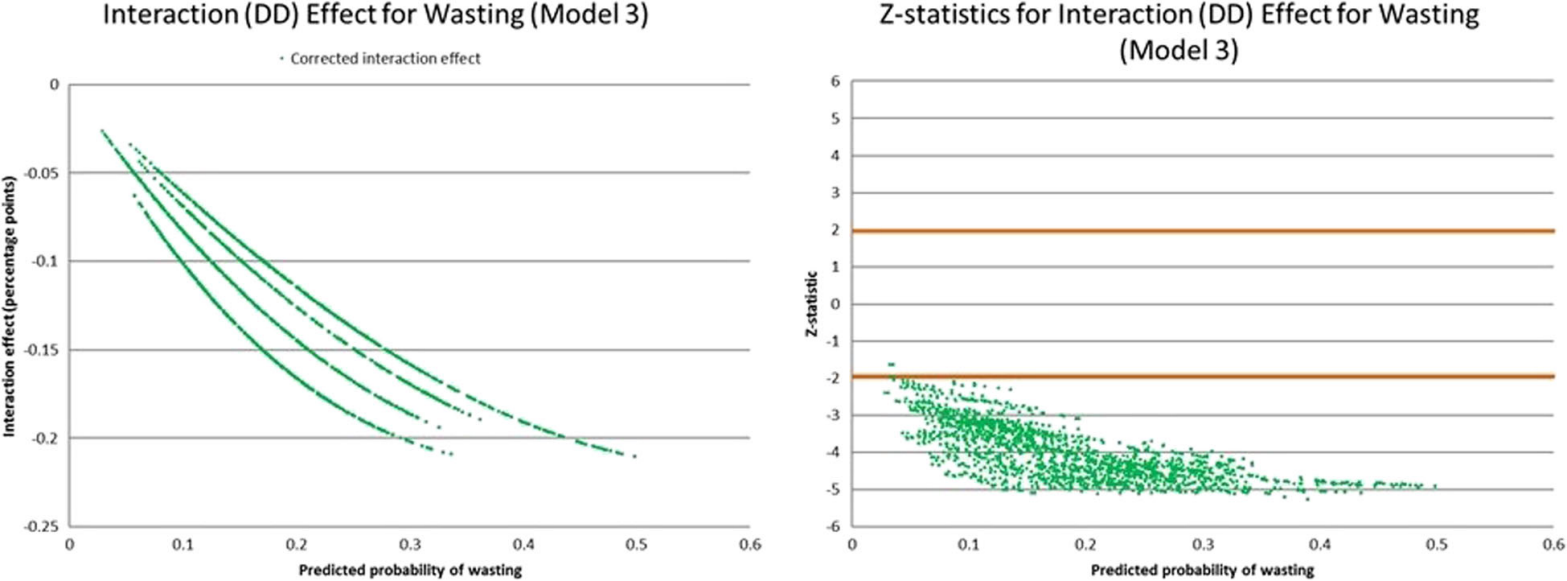
Graphic representation of the marginal effects for the interaction term (double-difference) for wasting, Model 3 with select covariates. Left panel is the interaction effect and right panel is z-statistics for individual children

Table 4 shows the marginal effects for the interaction term, which is the estimate of the double-difference for the probability of wasting. Model 1 is without covariates, Model 2 has all environmental, household, and child covariates, and Model 3 has select covariates based on their significance in Model 2 (*p* < 0.05). The results suggest that, ceteris *paribus*, if children who were exposed to the Boko Haram insurgency had not been exposed, the likelihood of wasting would decrease by 13 percentage points. Fig. 4 shows these results graphically, illustrating that there is some variance in the marginal effects of the conflict on individual children, but that the overall pattern is similar. The results are statistically significant for nearly all the individual children (mean z-statistic = − 4.2). Covariates are shown in Additional file 1: Table S1.

**Table 4:** Corrected marginal effects for the interaction term (double-difference) for wasting. Model 1 has no covariates, Model 2 includes all environmental, household, caregiver, and child covariates, Model 3 has select covariates based on significance in Model 2 (*p* < 0.05)

	Wasting Model 1				Wasting Model 2				Wasting Model 3			
	Mean	Std. Dev.	Min	Max	Mean	Std. Dev.	Min	Max	Mean	Std. Dev.	Min	Max
Interaction term (DD)	−0.157	0.000	−0.157	−0.157	−0.138	0.048	−0.224	− 0.019	−0.134	0.044	−0.210	− 0.026
Std. Err.	0.034	0.000	0.034	0.034	0.034	0.008	0.008	0.075	0.031	0.007	0.010	0.046
Z-statistic	−4.573	0.000	−4.573	−4.573	−3.979	0.721	−5.406	−1.357	−4.205	0.692	−5.262	−1.626

## Discussion

This study aimed to estimate the effect of the Boko Haram insurgency in Northeast Nigeria on childhood wasting. Visualization of common trends from 1990 through 2013 showed that wasting is plausibly linked to the insurgency, thus justifying the use of a double-difference methodology. The differences in outcomes among two groups – those in conflict and non-conflict areas and in two periods – before and after the start of the conflict – were compared using household data from the Demographic and Health Surveys.

Results suggest that if children who were affected by the Boko Haram insurgency in Northeast Nigeria had not experienced the conflict, they would have a mean WHZ 0.49 standard deviations higher than it is. For children already in the range of normal weight-for-height (generally − 1 to + 2 SD from the reference population), this would not be a large difference. However, for at-risk children and those already suffering from malnutrition, this increase would mean better short-term health, increased resistance to other illnesses, and reduced risk of mortality.

This analysis also estimates with a high degree of significance that the likelihood of childhood wasting would have been 13 percentage points lower in the absence of conflict in Northeast Nigeria. This too would be an important difference, bringing the proportion down from 23% to 10% - close the average for all of West Africa (9%) [29, 30]. This would reduce the proportion of children at risk of dying from malnutrition and co-morbidities and would help ensure a healthier and more productive adult population in the future.

How the conflict affected child nutrition is not fully known, but news reports from around the time of the 2013 survey give a sense of violence and lawlessness in the conflict areas. There are reports of indiscriminate executions of ordinary people, suicide attacks, and the destruction of schools and entire towns [31]. Men were fleeing forced conscription by Boko Haram and civilian groups formed to fight the insurgents [31]. Reports from the Famine Early Warning Systems Network illustrate growing food insecurity during this time [32]. In March 2013, Yobe, Borno, Adamawa, and Taraba (a non-conflict state) were considered “stressed” due to population displacement and below average harvest yields [32]. Security measures such as checkpoints reduced population movements and increased transportation costs [32]. Traders and their customers were concerned about their safety in markets, which reduced food stocks and overall market functionality [32]. By the time the state of emergency was declared in May, Yobe and Borno states were elevated to “crisis” stage as poor households faced depleted food stocks and acute food insecurity [32]. This marked the start of a worsening humanitarian crisis characterized by fear, displacement, disrupted services such as health care, schools, and maintenance of infrastructure, and limited access by local and international humanitarian assistance organizations, especially to more remote areas [33]. This led to a nutrition crisis as food insecurity increased and childhood illnesses exacerbated by malnutrition often went untreated [33].

The significant covariates in the regression models also offer insight into what factors contributed to child health in this setting. For example, while urban/rural differences were not significant, the presence of Maiduguri in the conflict areas may have mitigated the effects of violence. This may reflect an urban health advantage for Maiduguri, for example through overall better water and sanitation infrastructure (it is estimated that in 2013, 91% of Nigeria’s urban population had access to improved water supply and 73% to improved sanitation facilities), better access to healthcare, more robust food systems, etc. [34, 35]. The rainy season was more protective for mean z-scores, while the dry season was more protective for likelihood of wasting. This could be due to different seasonal dynamics affecting the whole child population as opposed to the most vulnerable. A healthy child may gain weight if food security improves in the rainy season, e.g. from a household garden [36]. But a child who is already thin may lose weight by the end of the rainy season if last year’s stores are low and this year’s harvest is not yet in (“the lean season”). This is consistent with findings that mortality often peaks at the end of the rainy season [5].

If the mother had some education, her children have significantly better weight-for-height and wasting outcomes, consistent with other studies [37]. In this population, among mothers with some education, the majority (56%) have only at least some primary schooling, which suggests even minimal education can make a difference in child health outcomes. Age of the child is significant for WHZs, indicating that children are more vulnerable to food insecurity as they are weaned.

There are several limitations with this study. There is likely unidentified confounding where certain factors influence both conflict and malnutrition outcomes. Additionally, Boko Haram is not the only cause of violence in the Northeast and there could be other unobserved dynamics that affect the results. The displacement or migration history of households is not known from these data, but interaction between the two groups is likely. It is equally possible that those with means or the most vulnerable left the Northeast altogether or moved to Maiduguri, making the direction of any bias difficult to determine [38]. Finally, six clusters in the conflict states could not be surveyed in 2013 due to security concerns [39]. Again, whether this biases the results and if so, in which direction, is not determinable because we do not know the status of those children.

## Conclusions

Children in poor areas of the world already face enormous risks. When conflict erupts, an already fragile existence can be made even more unstable. Malnutrition is a complex condition which can be brought on or exacerbated in many ways related to conflict from increased food prices to a case of dysentery going untreated because health care facilities have shut down. Thus, this research does not purport that conflict is the only challenge for young children in the developing world, but it illustrates quantitatively that exposing them to armed conflict increases the risk of morbidity and mortality in ways other than trauma.

The results of this study underscore the importance of programs and policies which aim to improve the nutritional status of children in conflict areas. This requires multi-level interventions, an integrated approach, and the flexibility to address immediate needs while working toward long-term solutions. For example, individuals most vulnerable to malnutrition (young children, but also pregnant and lactating women, the elderly, those with chronic illnesses) must be prioritized for nutritional support interventions. At the same time, community-wide issues such as access to health care and the provision of clean water must also be addressed. Similarly, even as acute needs are being met, policies and programs must aim to build resilience in households and communities through education, economic empowerment, improved food security, and good governance.

## Additional file

Additional file 1: Table S1. Covariates for the logistic regression model for wasting. (DOCX 30 kb)

## Abbreviations

CI: Confidence interval
DD: Double-difference
DHS: Demographic and Health Surveys
SD: Standard deviation
UNICEF: United Nations Children’s Fund
WHO: World Health Organization
WHZ: Weight-for-height z-score

## References

[1] Toole M, Waldman R (1997). The public health aspects of complex emergencies and refugee situations. Annu Rev Public Health.

[2] Johns Hopkins Bloomberg School of Public Health and the International Federation of Red Cross and Red Crescent Societies. The Johns Hopkins and Red Cross Red Crescent Public Health Guide in Emergencies*.* 2008.

[3] Moss WJ, Ramakrishnan M, Storms D, et al. Child health in complex emergencies. Geneva: Bulletin of the World Health Organization; 2006. 10.2471/blt.04.019570 PMC2626512 16501716

[4] World Health Organization (2014). Children: reducing mortality.

[5] Coghlan B, Brennan RJ, Ngoy P (2006). Mortality in the Democratic Republic of Congo: a nationwide survey. Lancet.

[6] Agadjanian V, Prata N (2003). Civil war and child health: regional and ethnic dimensions of child immunization and malnutrition in Angola. Soc Sci Med.

[7] Brown V, Guerin PJ, Legros D, Paquet C, Pécoul B, Moren A (2008). Research in complex humanitarian emergencies: the Médecins Sans Frontières/epicentre experience. PLoS Med.

[8] Internal Displacement Monitoring Centre. Nigeria: Fragmented response to internal displacement amid Boko Haram attacks and flood season. Oslo: Norwegian Refugee Council; 2013.

[9] Pérouse de Montclos M-A (2014). Nigeria’s interminable insurgency? Addressing the Boko Haram crisis.

[10] Reinert M, Garçon L, Pérouse de Montclos M-A (2014). Boko Haram: a chronology. Boko Haram: Islamism, politics, security and the state in Nigeria. Vol 2.

[11] ACAPS (2015). Secondary data review – 24 august 2015 Northeast Nigeria conflict – Adamawa, Borno, Gombe, and Yobe states.

[12] Mohammed K, Pérouse de Montclos M-A (2014). The message and methods of Boko Haram. Boko Haram: Islamism, politics, security and the state in Nigeria Vol 2.

[13] The DHS Program. Demographic and Health Surveys. http://www.dhsprogram.com/.

[14] Aliaga A, Ren R (2006). Optimal sample sizes for two-stage cluster sampling in demographic and health surveys.

[15] UNICEF. Nutrition in emergencies. https://www.unicef.org/nutrition/training/. Accessed Nov 2014.

[16] Esri. World Populated Places. 2011; http://www.arcgis.com/home/item.html?id=587c838521864164acd245ea03315006.

[17] Rutsein SO, Johnson K. The DHS Wealth Index. Calverton: The DHS Program; 2004.

[18] WHO/UNICEF Joint Monitoring Programme for Water Supply and Sanitation (2006). Core questions on drinking-water and sanitation for household surveys.

[19] Gascón J, Vargas M, Schellenberg D (2000). Diarrhea in children under 5 years of age from Ifakara, Tanzania: a case-control study. J Clin Microbiol.

[20] Black RE, Victora CG, Walker SP (2013). Maternal and child undernutrition and overweight in low-income and middle-income countries. Lancet.

[21] Buvinic M, Das Gupta M, Casabonne U, Verwimp P (2013). Violent conflict and gender inequality: an overview. World Bank Res Obs.

[22] Brewer M, Crossley TF, Joyce R (2013). Inference with difference-in-differences revisited.

[23] Dickson L, Pender M (2013). Do in-state tuition benefits affect the enrollment of non-citizens? Evidence from universities in Texas. Econ Educ Rev.

[24] Norton EC, Wang H, Ai C (2004). Computing interaction effects and standard errors in logit and probit models. Stata J.

[25] Lechner M (2010). The estimation of causal effects by difference-in-difference methods. Foundations Trends Econometrics.

[26] World Food Programme and Centers for Disease Control and Prevention. A Manual: Measuring and Interpreting Malnutrition and Mortality. Rome: World Food Programme and Centers for Disease Control and Prevention; 2005.

[27] Garrett JM (2006). Svypxcat.

[28] Leroy JL (2011). zscore06: Stata command for the calculation of anthropometric z-scores using the 2006 WHO child growth standards.

[29] World Health Organization, United Nations High Commissioner for Refugees, International Federation of Red Cross, World Food Programme (2000). The management of nutrition in major emergencies.

[30] UNICEF, World Health Organization, World Bank Group (2015). Levels and trends in child malnutrition: UNICEF – WHO – World Bank Group joint child malnutrition estimates: key findings of the 2015 edition.

[31] IRIN (2013). Updated timeline of Boko Haram attacks and related violence.

[32] Famine Early Warning Systems Network. Nigeria. http://www.fews.net/. Accessed Oct 2015.

[33] Watchlist on Children and Armed Conflict. “Who will care for us?” Grave violations against children in northeastern Nigeria. New York: Watchlist on Children and Armed Conflict; 2014.

[34] WHO/UNICEF Joint Monitoring Programme for Water Supply and Sanitation. Nigeria. https://washdata.org/data.

[35] Vlahov D, Galea S, Freudenberg N. The urban health “advantage.” J Urban Health. 2005;82(1):1-4. 10.1093/jurban/jti001. 10.1093/jurban/jti001 PMC3456628 15738341

[36] Galhena DH, Freed R, Maredia KM (2013). Home gardens: a promising approach to enhance household food security and wellbeing. Agriculture & Food Security.

[37] UNICEF (2015). Committing to child survival: a promise renewed. Progress report 2015.

[38] Akresh R, Lucchetti L, Thirumurthy H (2012). Wars and child health: evidence from the Eritrean–Ethiopian conflict. J Dev Econ.

[39] National Population Commission (Nigeria) and ICF International. Nigeria Demographic and Health Survey 2013. Abuja, Nigeria, and Rockville, Maryland, USA: National Population Commission (Nigeria) andICF International; 2014.

[40] Nigeria Watch. http://www.nigeriawatch.org/. Accessed November 2015.

