# Supplementary material for: The impact of the Boko Haram insurgency in Northeast Nigeria on childhood wasting: a double-difference study
Source: Confl Health. 2018 Jan 24;12:6. doi: 10.1186/s13031-018-0136-2 (PMC5782364; doi:10.1186/s13031-018-0136-2)
Supplement: Additional file 1: Table S1. — Covariates for the logistic regression model for wasting. (DOCX 30 kb) [file 13031_2018_136_MOESM1_ESM.docx]

## The Impact of the Boko Haram Insurgency in Northeast Nigeria on Childhood Wasting: A Double-Difference Study

**Additional File 1**

*Covariates for the logistic regression model for wasting (Table AF1)*

Overall, the effect of month of interview is more protective in the dry months (February decreased odds of 43%) than in the wetter months (August decreased odds of 34%) at p < 0.05. Higher altitude increases the odds slightly. Living in Maiduguri decreased the odds of wasting by 63% compared to living elsewhere. Both the mother’s and her partner’s education are significant in the final model with no formal education increasing the odds of wasting by 22%. If the mother is Muslim, the odds increase by 34%. The age and sex of the child is not significant for this outcome.

|  |  | **Wasting Model 1** | | | **Wasting Model 2** | | | **Wasting Model 3** | | |
| --- | --- | --- | --- | --- | --- | --- | --- | --- | --- | --- |
|  |  | OR | p-value | Std. Err. | OR | p-value | Std. Err. | OR | p-value | Std. Err. |
| States |  |  |  |  |  |  |  |  |  |  |
|  | Conflict states | *ref* |  |  | *ref* |  |  | *ref* |  |  |
|  | Non-conflict states | 1.760 | 0.000 | 0.233 | 1.930 | 0.000 | 0.289 | 1.890 | 0.000 | 0.258 |
| Period |  |  |  |  |  |  |  |  |  |  |
|  | 2008 | *ref* |  |  | *ref* |  |  | *ref* |  |  |
|  | 2013 | 1.380 | 0.023 | 0.194 | 1.734 | 0.004 | 0.329 | 1.640 | 0.007 | 0.298 |
| Month | |  |  |  |  |  |  |  |  |  |
|  | February |  |  |  | 0.586 | 0.002 | 0.101 | 0.571 | 0.001 | 0.099 |
|  | March |  |  |  | 0.547 | 0.000 | 0.057 | 0.548 | 0.000 | 0.053 |
|  | April |  |  |  | 0.657 | 0.001 | 0.081 | 0.659 | 0.000 | 0.075 |
|  | May |  |  |  | 0.636 | 0.000 | 0.067 | 0.644 | 0.000 | 0.065 |
|  | June |  |  |  | 0.640 | 0.000 | 0.079 | 0.626 | 0.000 | 0.071 |
|  | July |  |  |  | 0.614 | 0.000 | 0.077 | 0.606 | 0.000 | 0.071 |
|  | August |  |  |  | 0.665 | 0.022 | 0.118 | 0.661 | 0.009 | 0.104 |
|  | September |  |  |  | 0.970 | 0.827 | 0.136 | 0.980 | 0.877 | 0.129 |
|  | October |  |  |  | 0.759 | 0.085 | 0.121 | 0.753 | 0.058 | 0.112 |
| Altitude (m) | |  |  |  |  |  |  |  |  |  |
|  | Altitude |  |  |  | 1.003 | 0.003 | 0.001 | 1.003 | 0.002 | 0.001 |
|  | Altitude squared |  |  |  | 1.000 | 0.003 | 0.000 | 1.000 | 0.002 | 0.000 |
| Urban/Rural | |  |  |  |  |  |  |  |  |  |
|  | Rural |  |  |  | *ref* |  |  |  |  |  |
|  | Urban |  |  |  | 1.185 | 0.471 | 0.278 |  |  |  |
| Urban population | |  |  |  |  |  |  |  |  |  |
|  | 50 - 100,000 |  |  |  | 1.000 | (omitted) |  | 1.000 | (omitted) |  |
|  | 100 - 500,000 |  |  |  | 0.708 | 0.173 | 0.179 | 0.701 | 0.145 | 0.170 |
|  | > 1 million |  |  |  | 0.360 | 0.001 | 0.109 | 0.365 | 0.000 | 0.099 |
|  | Not urban |  |  |  | 1.000 | (omitted) |  | 0.847 | 0.427 | 0.177 |
| Wealth |  |  |  |  |  |  |  |  |  |  |
|  | Wealthiest 3 quintiles |  |  |  | *ref* |  |  |  |  |  |
|  | Poorest 2 quintiles |  |  |  | 0.969 | 0.767 | 0.104 |  |  |  |
| Water source | |  |  |  |  |  |  |  |  |  |
|  | Unimproved |  |  |  | *ref* |  |  |  |  |  |
|  | Improved |  |  |  | 1.050 | 0.553 | 0.087 |  |  |  |
| Toilet type | |  |  |  |  |  |  |  |  |  |
|  | Unimproved |  |  |  | *ref* |  |  |  |  |  |
|  | Improved |  |  |  | 1.008 | 0.925 | 0.090 |  |  |  |
| No. people in household | |  |  |  |  |  |  |  |  |  |
|  | No. people |  |  |  | 0.980 | 0.424 | 0.024 |  |  |  |
|  | No. people squared |  |  |  | 1.000 | 0.690 | 0.001 |  |  |  |
| No. under fives in household | |  |  |  |  |  |  |  |  |  |
|  | No. under fives |  |  |  | 1.032 | 0.792 | 0.123 |  |  |  |
|  | No. under fives squared |  |  |  | 0.998 | 0.918 | 0.017 |  |  |  |
| Mother's occupation | |  |  |  |  |  |  |  |  |  |
|  | Wage employment |  |  |  | *ref* |  |  |  |  |  |
|  | Subsistence/own activities |  |  |  | 1.062 | 0.463 | 0.087 |  |  |  |
| Partner's occupation | |  |  |  |  |  |  |  |  |  |
|  | Wage employment |  |  |  | *ref* |  |  |  |  |  |
|  | Subsistence/own activities |  |  |  | 1.043 | 0.612 | 0.086 |  |  |  |
| Mother's education | |  |  |  |  |  |  |  |  |  |
|  | Some education |  |  |  | *ref* |  |  | *ref* |  |  |
|  | No formal education |  |  |  | 1.262 | 0.017 | 0.123 | 1.221 | 0.031 | 0.113 |
| Partner's education | |  |  |  |  |  |  |  |  |  |
|  | Some education |  |  |  | *ref* |  |  | *ref* |  |  |
|  | No formal education |  |  |  | 1.235 | 0.007 | 0.096 | 1.229 | 0.007 | 0.093 |
| Mother's religion | |  |  |  |  |  |  |  |  |  |
|  | Other |  |  |  | *ref* |  |  | *ref* |  |  |
|  | Muslim |  |  |  | 1.320 | 0.079 | 0.208 | 1.342 | 0.041 | 0.193 |
| Child's age (months) | |  |  |  |  |  |  |  |  |  |
|  | Child's age |  |  |  | 1.001 | 0.853 | 0.008 |  |  |  |
|  | Child's age squared |  |  |  | 1.000 | 0.073 | 0.000 |  |  |  |
| Child's sex | |  |  |  |  |  |  |  |  |  |
|  | Female |  |  |  | *ref* |  |  | *ref* |  |  |
|  | Male |  |  |  | 1.128 | 0.047 | 0.068 | 1.089 | 0.140 | 0.063 |
| Birth order | |  |  |  |  |  |  |  |  |  |
|  | Birth order |  |  |  | 0.938 | 0.141 | 0.041 |  |  |  |
|  | Birth order squared |  |  |  | 1.006 | 0.078 | 0.004 |  |  |  |
| Intercept | |  |  |  |  |  |  |  |  |  |
|  | Constant | 0.214 | 0.000 | 0.019 | 0.140 | 0.000 | 0.064 | 0.128 | 0.000 | 0.052 |

Table AF1: Odds ratios (OR), p-values, and standard errors for covariates of logistic regression for wasting. Model 1 has no covariates, Model 2 includes all environmental, household, caregiver, and child covariates, Model 3 has select covariates based on significance in Model 2 (p < 0.05).
